# The Effects of Auricular Electro-Acupuncture on Ameliorating the Dysfunction of Interstitial Cells of Cajal Networks and nNOSmRNA Expression in Antrum of STZ-Induced Diabetic Rats

**DOI:** 10.1371/journal.pone.0166638

**Published:** 2016-12-08

**Authors:** Huan Chen, Weijian Zhu, Jing Lu, Jinqing Fan, Luning Sun, Xiaoke Feng, Hao Liu, Zhaohui Zhang, Yongqing Wang

**Affiliations:** 1 Department of Acupuncture, The First Affiliated Hospital, Nanjing Medical University, Nanjing, Jiangsu, China; 2 Department of Pharmacy, The First Affiliated Hospital, Nanjing Medical University, Nanjing, Jiangsu, China; 3 Department of Traditional Chinese Medicine, The First Affiliated Hospital, Nanjing Medical University, Nanjing, Jiangsu, China; The University of Texas MD Anderson Cancer Center, UNITED STATES

## Abstract

**Backgroud:**

Interstitial cells of Cajal (ICCs) and nNOS play a crucial role in diabetic gastrointestinal dysmotility(DGD). Our previous study found that electro-acupuncture(EA) on ear point ‘stomach’ could repair the gastric dysrhythmias in rats induced by rectal distention(RD) after meal. However, little were known about the possible effect of auricular electro-acupuncture (AEA) on diabetic rats. Thus, we designed this study to investigate the effect of AEA on streptozotocin(STZ)-induced diabetic rats.

**Method:**

Forty male Sprague_Dawley (SD) rats were injected with STZ, at the end of 8th week after injection, animals were randomly divided into four groups and received 2 weeks-treatment(10 times) respectively: control group(CON,n = 10, no stimulation), sham auricular electro-acupuncture group(SEA,n = 10, low frequency EA on earlobes), auricular eletro-acupuncture group(AEA,n = 10, low frequency EA on ear point ‘stomach’), and ST-36 group(ST-36,n = 10, low frequency EA on ST-36). Gastrointestinal (GI) motility was measured by GI transit rate. ICCs(c-kit+ expression) in antrum were analyzed by Immunohistochemistry and western blotting. NO level in blood serum were detected by Griess Reagent, and nNOSmRNA expression in antrum were determined by Real-time PCR.

**Results:**

GI transit rate and ICCs(c-kit+ expression) in antrum of AEA group have the tendency to increase compared with CON group, but had no statistics difference (*P>0*.*05)*. nNOSmRNA expression in antrum of AEA group was dramatically increased compared with CON group (*P = 0*.*037)*.

**Conclusions:**

Low frequency EA on ear ‘stomach’ point could significantly up-regulate nNOS mRNA expression and ameliorate the ICCs networks partly in gastric antrum of STZ -induced diabetic rats, which may has benefits on regulating the GI motility.

## Introduction

Neuropathy is a common and costly complication of both type 1 and type 2 diabetes, whose prevalence is estimated to be about 8% in newly diagnosed patients and greater than 50% in patients with longstanding disease [1–2]. Gastrointestinal(GI) dysmotility as one of the most serious complication of diabetic gastrointestinal autonomic neuropathy[3], is often accompanied with symptoms like nausea, bloating, abdominal pain, diarrhea, constipation, delayed gastric emptying which leads to the impaired glycaemic control, and nutritional insufficiency[2, 4–7].

Interstitial cells of Cajal (ICCs) are specialized mesenchymal cells, playing a crucial role in GI motility. ICC were first discovered in the tunica muscularis of the GI tract by Ramon y Cajal in the 19^th^ century [8–9]. It has been verified that ICC networks originate slow wave intestinal pacemaker activity and mediate the input from the enteric motor neurons [10–11]. There are at least three separate functional groups of ICC exist: myenteric ICC (ICC-MY), intramuscular ICC (ICC-IM) and septa ICC (ICC-SEP)[12]. Among them, ICC-IM plays a key role in generating gastric slow waves in antrum, regulated by excitatory or inhibitory neurotransmitters [13]. ICCs could be identified by c-kit positive staining. C-kit is a tyrosine kinase, a 145 kD transmembrane glycoprotein, playing the very important role in the development of ICCs. Several studies have showed ICC networks were gradually disrupted in model animals with lacking c-kit (W/Wv) or kit ligand (Sl/Sld) [14] In clinic, losses of KIT expression and decreases in KIT-positive ICCs are associated with motility disorders and are correspondingly found in human GI diseases including diabetic gastroparesis, gallbladder dysmotility, slow transit constipation and others [12,15]. By now, as definite a marker, c-kit protein is looked as the gold standard in many studies to observe the change of ICCs in gut[14].

Nitric oxide (NO), a major inhibitory neurotransmitter, is released by nitrergic neurons in response to nerve stimulation and causes relaxation of the smooth muscle of the GI tract[16–17], synthesized by nitric oxide synthase (nNOS) detected in the myenteric plexus[18]. Thus, the absence or relative deficiency of nNOS leads to the impaired gastric accommodation and abnormal gastric emptying [11,19]. In addition, it also brought the intestinal dysmotility with the deficiency of NO[20]. Therefore, NO and nNOS are another critical roles for GI motility.

Acupuncture is a traditional Chinese therapy with more than two thousand years old and used to regulate GI disorders for longtime. Furthermore, symptoms of GI disorders appeared in diabetic gastroparesis such as nausea, vomiting, diarrhea, etc. could be relieved after needle stimulations [21–22]. One of the most important mechanisms was found that electro-acupuncture (EA) on ST36 could repair the injured ICC networks in gastrointestinal tract of diabetic rats[23–24].

Auricular acupuncture (AA), one of the most important branches of acupuncture, is also used in Europe and China with long history [25–26]. which looked as a diagnostic method and effective treatment of visceral diseases including functional dyspepsia (FD)[27–28]in clinic. Recently, Li H, et al have reported that low frequency auricular electro-acupuncture (AEA) on points in cavum conchae could improve the GI motility significantly in model rats [29–30].

Our previous research also found that electro-acupuncture on ear point ‘stomach’ in cavum conchae could repair the gastric dysrhythmias in rats induced by rectal distention(RD) after meal. The effects of AEA in those experiments could be blocked by atropine, suggested that AEA improving the GI motility via vagal pathways[31]. We hypothesized that the effect of low frequency AEA on improving the abnormal gastric motility came from restoring the ICCs networks disorders in antrum via vagal pathways. In order to verify the hypothesis, we designed this study to investigate possible effects of AEA on GI dysmotility in STZ-induced diabetic rats by measuring the percentage of GI transit, serum NO and the expression of ICCs and nNOSmRNA in gastric antrum.

## Material and Methods

### Ethics statement

All animal procedures performed in this study were approved by the Animal Care and Use Committee of Nanjing Medical University.

### Animal preparation

40 male Sprague_Dawley (SD) rats(9 weeks old)(Center of Experimental Animals, Nanjing Medical University, Nanjing, China), were housed in individual plastic cages. All cages with experimental animals were maintained in the institutional animal care facility under controlled temperature (24°C), humidity (40–50%) and light-dark cycle (12: 12-h), with free access to rodent chow and water.

### Modeling

Diabetic rats were prepared by intraperitoneal injection of streptozotocin (STZ, 55 mg kg−1) (Sigma Chemical, St. Louis, MO, USA) dissolved in 9 mmol citrate buffer, pH 4.0. Two rats died immediately after STZ injection. Blood glucose levels were examined at 72h after STZ injection. Animals exhibiting random blood glucose levels more than 16.6mmol/L were included as diabetic rats[32]. Animals do not reached the standard were eliminated. According to previous researches, diabetic gastroparesis will form after 6–8 weeks in STZ-induced diabetic rats [32–34]. We monitored the blood glucose two times per week, once the random blood glucose falls 16.6mmol/L, the animals should be eliminated. Extra rats were molded to make up a deficiency. At the end of 8th week after STZ-injection, animals were divided into four groups. They are the control group (CON,n = 10), sham auricular eletro-acupuncture group(SEA,n = 10), auricular eletro-acupuncture group(AEA,n = 10), and ST-36 group(ST-36,n = 10).

### Acupuncture treatment

Two stainless steel needles (Model Hua-tuo 0.5 mm diameter, Medical Appliance Factory Co.,Ltd, Suzhou, China) were inserted bilaterally at the stomach point(CO40)in the auricles of the rat and then punched through the auricular gristle for about 3mm and curved the pinpoints at the narcotized state. CO40 was located between the cymba conchae and cavumconchae[31]. Electrical stimulation was performed via the needles using a universal pulse generator (0.7–1.0mA,2 Hz, 20 min, Hanz, LH202H, China). This set of parameters was used in AEA studies successfully in improving gastric motility in our previous study[31].Sham-AEA was performed using the same parameters but via needles inserted at bilateral earlobes. In ST-36 group, stimulation was performed on the ST-36 points. ST36 point in the rat was located at 5mm below head of fibula under knee joint, and 2mm lateral to the anterior tubercle of the tibia [35]. A pair of stainless steel needles was inserted bilaterally at a depth of 3–5mm into the skin at ST36. The ST36 was electrically stimulated using the same pulse generator and same stimulation parameters as AEA except for the amplitude of 1.0–1.2mA. All the rats kept in consciousness during the EA stimulation, once/day, 5d/week, 2 weeks. Needles were inserted when the rats were anaesthetized by ether inhalation and electro-acupuncture treatment was performed after the animals awake. Rats in control group also received ether inhalation anesthesia, but no treatment. All the rats kept in consciousness during the EA stimulation, once/day, 5d/week for 2 weeks. Three rats died because of anesthesia (one in control group, the other two in Sham-AEA group and in ST-36 group respectively).

### Sample collection and processing

#### Serum indicators

Blood samples were obtained from orbital vein under anesthesia before acupuncture treatment. NO was detected at the beginning and the end of the acupuncture treatment by nitric oxide assay kit (Beyotime,China), separately.

#### Gastrointestinal transit studies

At the end of two weeks of acupuncture treatment, according to the protocol, all animals were fasted over 24h. After the fasting, four rats in each group were selected randomly. Each rat was given a gavage of Indian ink. One hour later, animals were euthanized, the cardia and pylorus were ligated and the entire GI tract was collected. The GI transit rate was calculated according to the following equation [29]:

GItransitrate(%)=thedistancethatIndianInkhadmigratedfrompylorus/thelengthofintestine×100%.

#### Immunohistochemistry analysis of ICC (c-Kit+)in gastric antrum

The antrum tissue specimens were immediately fixed by immersion in 4% paraformaldehyde for 24 h, and then processed for paraffin embedding in a vacuum and cut to a thickness of 4μm. Sections were de-paraffinized in xylene and hydrated in a graded solution of ethanol. After endogenous peroxidase activity was quenched with 3% hydrogen peroxide (H_2_O_2_) for 10 min and microwaved (750 W) for 5 min, then nonspecific binding was blocked by treatment with normal rabbit serum for 30 min at 37°C. The primary antibodies c-Kit (1:100, Santa Cruz Biotechnology, Santa Cruz, CA) was added to the sections in a moist chamber overnight at 4°C. The slides were washed three times in 0.01mol/l PBS (pH 7.2) and incubated with secondary antibody(HRP-linked polymer antimouse/rabbit IgG)(1:200,ZSGB-Bio,China)for 30 min at 37°C. After washing in PBS three times, the localization of target protein was visualized by incubating the sections for 10 min in freshly prepared 3,3-diaminobenzidine(DAB)solution. The slides were washed again, counterstained in hematoxylin, and then dehydrated. Specificity of the antibody was confirmed by negative control (absence of primary antibody). The slides Positive immunostaining was evaluated at a magnification of x200, using an Olympus FV500 optical microscope (Olympus, Tokyo, Japan).

#### Western blotting analysis

Fresh-frozen antrum specimens were homogenized in extraction buffer, and were centrifuged at 12,000 g for 10 min at 4°C, and protein concentration in the supernatant was quantified by the bicinchoninic acid (BCA) method. Later, equivalents of 40μg of extracted proteins were separated using 12% SDS-PAGE, and the separated proteins were then transferred electrophoretically onto PVDF membranes. After blocking nonspecific binding sites with 5% nonfat dry milk in Tris·HCl-buffered saline (TBS) for 1 h, the membranes were then incubated with primary antibodies to c-Kit (1:200,sc-168,Santa, CA.), respectively, overnight at 4°C. Anti-rat GAPDH (1:500,AP0063, Bioworld Technology, Inc.) served as the internal control. After that, the membranes were washed in TBST (TBS with 0.1% Tween-20) for three times and incubated with HRP-linked secondary antibody (1:5,000,Goat anti-Rabbit IgG-HRP,Bioworld Technology, Inc.) for 1 h at room temperature. Detection of protein was achieved by ECL reagents, and the blot was subjected to autoradiography. A semiquantitative measurement of the band intensity was performed by Quantity One (ChemiDoc XRS+ System, Bio-RAD,Inc.)

#### nNOSmRNA expression in rat gastric antrum

nNOSmRNA expression levels were measured by real-time quantitative reverse-transcription PCR. Total RNA was extracted using the TRIzol reagent, and RNA samples were reversely transcribed into cDNA, according to the manufacturer’s instructions. The cDNA was subsequently diluted in RNase-free dH_2_O 20μl and stored at -20°C. The specific primers(NCBI Reference Sequence: NM_052799)is the rat nNOS (Forward: 5‘CGGCTGTGCTTTAATGGAGAT 3’, Reverse: 5‘GAGGAGACGCTGTTGAATCG 3’). PCR was performed as described by the manufacturer using the SYBR Green PCR Master Mix(PrimeScript™ RT reagent Kit: Takara Bio Inc, Japan). The reaction was performed in triplicate in total volume of 20μl(containing 10μl SYBR Green/enzyme reaction mix, Forward primer 1μl, Reverse primer1μl, cDNA 1μand ddH_2_O 7μl). The PCR conditions were 95°C for 15 min first, and 95°C for 10s, then followed by 40 cycles by 56°C for 20s and 72°C for 20s. Real-time detection was performed on an BIO-RAD CFX Manager (Bio-Rad Laboratories, Inc, US).Fluorescence values of SYBR Green dye, representing the amount of product amplified at that point in the reaction, were recorded in real time at both the annealing step and the extension step of each cycle. The amplification was followed by a melting curve analysis, which was applied to all reactions to ensure homogeneity of the amplification product. Melting curve analysis was performed by increasing the temperature by 0.5°C increments from 65°C to 95°C and measuring the fluorescence at each temperature for a period of 5s. The transcript level of each specific gene was normalized to the housekeeping gene GAPDH(Rat *GAPDH* Forward: 5‘GACATAAAGGAGAAGCTGTGC 3’,Reverse: 5‘CATGATGGAGTTGAAGGTGGT 3’,NCBI Reference Sequence: NM_017008).

### Statistical analysis

Data were presented as means ± standard deviation. Differences among groups were examined using one-way ANOVA, followed by Student-Newman-Keuls test. Analyses were performed with SPSS software version20.0; *P*< 0.05 was considered to be statistically significant.

## Results

### General condition

Normal rats are alert and lively, with smooth, dense, and shiny hair and normal urination and defecation. Diabetic model rats began to exhibit highly raised blood glucose (with random blood glucose > 16.6mmol/L), polydipsia, polyphagia, diuresis, and loose stool 72h after STZ-injection, and significantly lowered average weight, loose stool, lassitude, bad-response, emaciation, and thinning hair 1 week after the injection. At the 4th week, most rats were of reduced food intake and loose stool. And at the 6thweek, most rats were of significantly reduced food intake, emaciation, lassitude, bad-response, thinning hair, swollen stomachs and loose stool, other than polydipsia, polyphagia, and diuresis.

### GI transit rate

**Fig 1:**
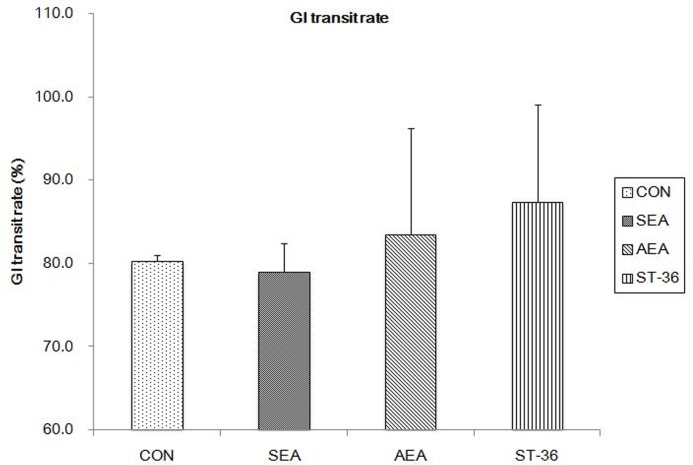
GI transit rate(n = 4). No significant difference were found among CON group, SEA group,AEA group and ST-36 group(P>0.05).

As shown in Fig 1, GI transit rate between CON group (80.23±0.70) and SEA group(79.0±3.46), have no obvious difference(P = 0.848). AEA group(83.45±12.76) and ST-36 group(87.33±11.80) both Higer compared with CON group, however, have no statistical differences(P = 0.616 and P = 0.279).

### NO change in peripheral blood

**Fig 2:**
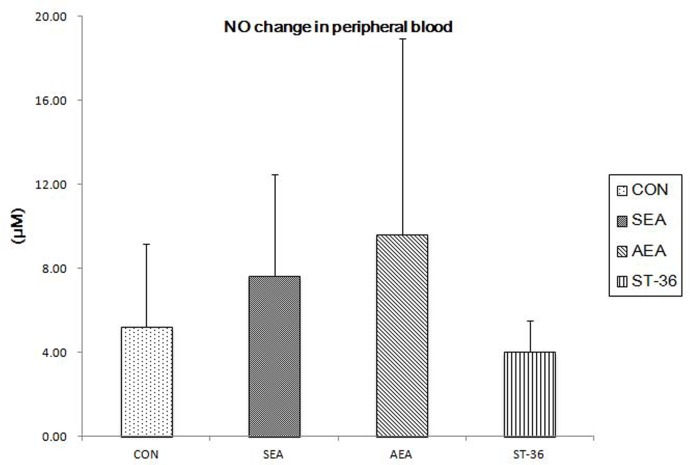
NO released in rats serum(n = 8). There was no significant difference of NO content in rats serum between CON group,SEA group,AEA group and ST-36 group (P>0.05).

As shown in Fig 2, NO in rats serum from SEA group(7.61±4.86) and AEA group(9.61±9.31) were released more compared with CON group(5.21±3.98), however, these differences were not significant(P = 0.829 and P = 0.766)There have no obvious difference between CON group and ST-36group(5.21±3.98vs4.03±1.49,P = 0.957).

### Effects of acupuncture on ICC morphological change(c-Kit+) in antrum tissue

**Fig 3:**
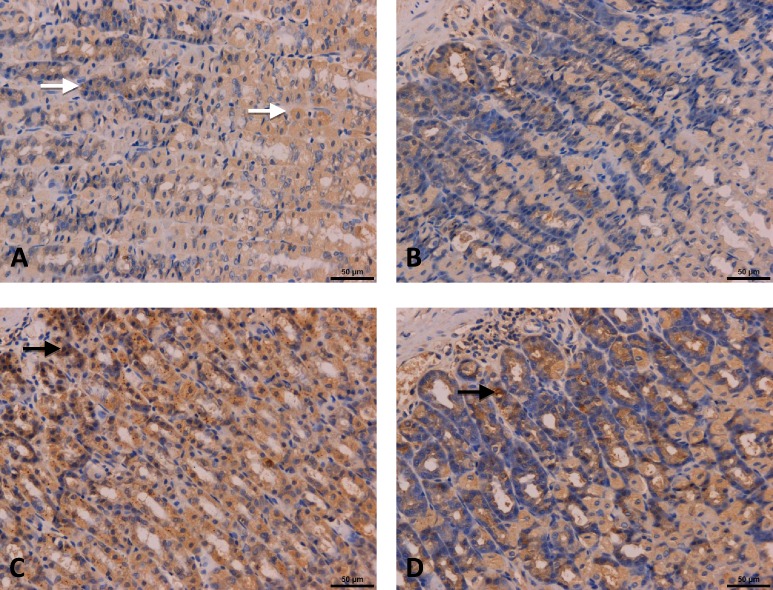
Effects of acupuncture on antral ICC networks in diabetic rats. **(n = 3)** ICCs were identified by c-kit positive staining (claybank). In diabetic rat from CON group(Fig 3-A), ICCs scatter with slightly staining in both submucous and muscle layers (white arrow). The c-kit+ cellular networks were incomplete in SEA group(Fig 3-B), and c-kit+ expression was dramatically increased in antrum of AEA group(Fig 3-C, dark arrow), and the distribution of ICC becomes normal, as well as that in EA ST-36 group(Fig 3-D, dark arrow).

ICCs were identified by c-kit positive immunolabeling. There were throughout the submumous and muscle layers with irregular accumulation of cells between the muscle layers in diabetic rats of CON group (Fig 3A, white arrow). Only a few number of c-kit positive cells were found in SEA group(Fig 3B). In contrast, c-kit positive was dramatically increased in antrum in AEA group(Fig 3C, dark arrow), and the distribution of ICC becomes normal, as well as that in EA ST-36 group(Fig 3D, dark arrow).

### Effects of acupuncture on c-Kit protein expression in antrum tissue

**Fig 4:**
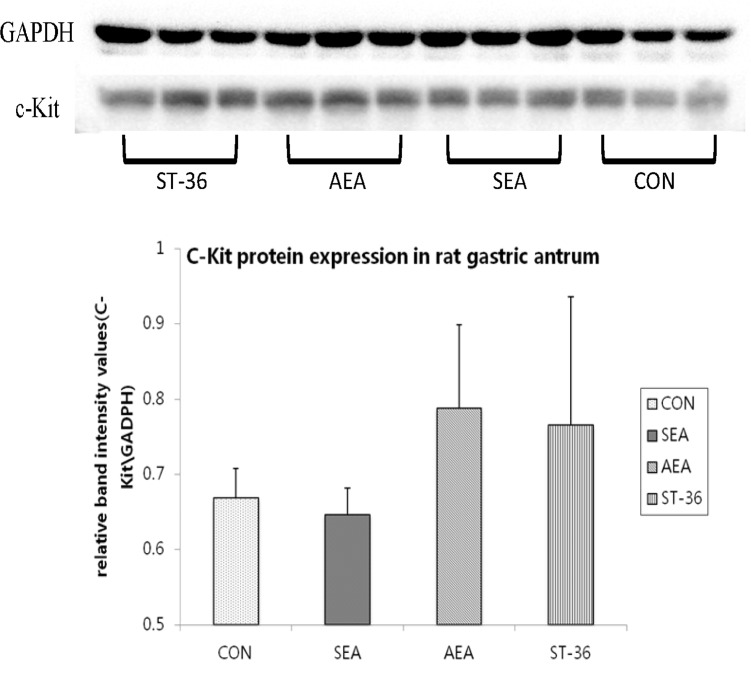
c-Kit protein expression in antrum Tissue(n = 3). Compared with CON group, c-kit expression in antrum were increased in AEA group and ST-36 group, however, these change haven’t statificantly(P>0.05).

Western blotting was applied for quantitative analysis of c-kit expression (Fig 4). As well as Histopathological change (Fig 3), c-kit+ expression in AEA group(0.79±0.11) and ST-36 group (0.77±0.17) from antrum were both improved compared with CON group(0.67±0.04), and SEA group(0.65±0.04)was not apparently different with CON group. However, there’s no significantly difference among these four groups(P = 0.408).

### Effects of acupuncture on nNOSmRNA expression in antrum tissue

**Fig 5:**
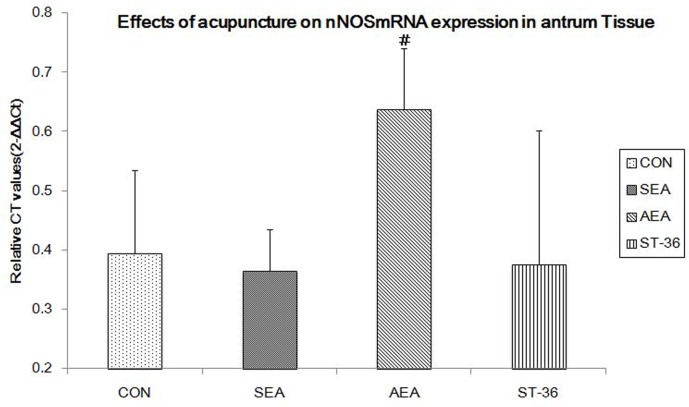
nNOSmRNA expression in rats antrum(n = 4). nNOSmRNA expression in rats antrum from AEA group was dramatically increased compared to CON group(p = 0.037), SEA group (p = 0.022)and ST-36group(p = 0.027). #compared with CON, SEA, and ST-36 group, P<0.05.

As shown in Fig 5, in rat gastric antrum, a significant reduction in nNOSmRNA expression was observed in CON group compared to AEA group(0.39±0.14 VS 0.64±0.10,p = 0.037). There were significant difference between AEA group and SEA group(0.64±0.10 vs0.36±0.07,p = 0.022),AEA group and ST-36 group(0.64±0.10 VS 0.37±0.23,p = 0.027).

## Dicussion

The present study demonstrated that low frequency electro-acupuncture on auricular point ‘stomach’ (CO4) could improve the GI motility, significantly increase nNOS mRNA expression and stimulate cytothesis of ICCs in gastric antrum in STZ-induced diabetic rats.

It has well known that ICCs networks originate slow wave intestinal pacemaker activity and mediate the input from the enteric nervous systems [10–11,14] and play crucial roles in the physiological function of smooth muscle tissues in GI tract [13]. It also was believed that ICCs decreased number and/or structural dramatically injured with hyperglycaemia ultimately contribute to GI dysmotility [11]. On the other hand, ICCs dysfunction without ICCs loss may lead to abnormal electrical slow wave activity in acute hyperglycemia and cause the abnormal GI transit as well[11]. Therefore, gastroparesis derived from abnormal gastric slow waves was always found in diabetic caused by the loss or dysfunction of ICCs networks [11,36].

In this study, lower GI transit of rats in the control group was showed because of the ICCs dysfunction in hyperglycemic circumstance. The expression of c-kit protein in gastric antrum of STZ rats increased better in AEA group than that of the other three groups, suggesting the low frequency AEA could repair the injured ICCs very well. Further, the result of immunohistochemistry in our study also verified the effect of AEA on repairing the injured ICCs. Also, the lower GI transit was concordantly repaired obviously after AEA stimulations compared with that in control group. The results suggested that AEA could improve the GI motility in STZ-induced diabetic rats through repairing the ICCs dysfunction in gastric antrum. Interestingly, our previous research found that AEA on ‘stomach’ point could relieve gastric dysrhythmias induced by RD in rats [31], implied strongly that AEA could improve abnormal gastric slow waves through regulating the ICCs function in antrum of control rats. In this study, the results confirmed that AEA could repair the injured ICCs in anturm and improve the GI motility in STZ induced diabetic rats.

However, we haven’t known very well that what the pathway was the effect of AEA on repairing the injured ICCs. Therefore, we measured the expression of nNOSmRNA in gastric antrum and serum NO concentration in blood in STZ induced diabetic rats. It has well known that ICCs in GI tract are considered to perform a pacemaker function for GI motor activity by mediating excitatory (cholinergic) and inhibitory (nitrergic) neurotransmissions[20].

The main effect of nitrergic signaling was controlling gastric accommodation and pyloric relaxation at physiological status. The impairment in nitrergic relaxation was always seen in diabetes rats because of neuronal loss or dysfunction of nitrergic nerves [37]. NO, catalyzed by nNOS, had both a direct and indirect influence on neurotransmission and initiates smooth muscle relaxation. NO also could restore gastric dysmotility by increasing blood flow in stomach [38]. Several studies have reported that the absence of nNOS was related to the loss of ICCs and caused gastroparesis [39] and the possible mechanism involved the nNOS abnormalities of the myenteric plexus in diabetic gastroparesis[37,40–41]. In this study, the expression of nNOS mRNA in antrum remarkably increased after AEA stimulation compared with that of control group, indicating nNOS mRNA was restored after low frequency electro-acupuncture in the auricular point ‘stomach’ in STZ rats. Although no significant difference of serum NO concentration was observed in all the groups, the serum NO concentration in AEA group was also higher than that in the other three groups. The results suggested that AEA could repair the ICCs dysfunction through improving the function of nitrergic nerve in STZ induced diabetic rats.

In our previous study, the effect of AEA on ameliorating the injuried gastric slow wave induced by RD was blocked by atropine, suggesting that the effect of AEA was mediated via the vagal pathways [31]. Terry L. Powley has demonstrated the structure of NOS+ fibers as one phenotype of vagal afferents formed close appositions with the ICCs chains in the smooth muscle and the longitudinal and circular muscle wall of the antrum with terminal neurites of Intramuscular arrays (IMAs) [33]. It has reported that ICC-IM was closely associated with not only enteric motor nerves but also vagal afferent nerves supplying mechanoreceptors to the smooth muscle layers of the GI tract [12,33,42]. Therefore, vagal nerves play crucial role in connecting neurotransmitters (nitrergic) with ICCs GI tract. As Henry et al. has pointed out the afferent projection from the auricular branch of vagal nerve (ABVN) to the nucleus of tractus solitaries (NTS) in brain, which had extensive connection with many brain area including dorsal motor nucleus of vagus nerve (DVMN), etc[43]. ABVN played a key role on the effect of auricular acupuncture on regulating organs[44]. The significant increase of the nNOS mRNA expression in antrum by AEA in this study suggested that the key mechanism of the effect of AEA on restoring function of nitrergic nerve in STZ rats was built on the regulation of vagal nerves by AEA. Many reports have showed that it was the good way to regulate the dysfunction of some organs such as heart, lung and stomach, etc. through stimulating the area of cavum conchae densely distributed ABVN, but not other areas on auricle, because NTS could mediate many reflexes including respiratory and GI reflexes to regulate organ functions[25,44].This is the reason why there was no significant change of the expression of nNOS mRNA in antrum in sham AEA group by stimulating earlobe in this study. According to our previous as well as the result of this study, we hypothesized that low-frequency AEA may improve the gastrointestinal function of diabetic rats through the vagus nerve. Further study is needed to prove our hypothesis.

In this study, we prepared type 1 diabetic rats induced by STZ injection. And acupuncture treatments were applied in 8th weeks. Research has shown diabetic rats exhibited significant delayed gastric empty 8 wk after STZ injection. Selim C et.al[34] found that the depletion of nNOS in nitrergic nerve fibers of gastric tissues appeared from the early stages(4th week) of STZ-induced diabetes, and structural damage of the nitrergic nerve semerged from the 8th week. Wang et al[45] showed that the ICCs density were dramatically reduced in the antrum of the STZ-diabetic rats in 8th weeks after single injection of STZ, which was associated with ultrastructural change in nerves and the loss of enteric nerves. The ICC-MY and ICC-IM were reduced in antral muscle (especially the distal antrum) of 11 no-obese diabetic mice out of the 12[46].

Another study found that there was a decrease in NOS expression in the antrum, but not duodenum, ileum, and colon of diabetic rats three months after STZ injection[41]. Furthermore, gastric emptying and the protein level of nNOSα(the only functional isoform of nNOS in gastric antrum muscle strip), 9 weeks after STZ injection, was significantly decreased [47].Generally, the 8th week was the decisional time point for gastroparesis induced by injured ICCs networks after STZ injection in the diabetic rats.

In this study, the expression of nNOS mRNA in antrum did not increase significantly in EA group compared with that in control group, which suggested that the effect of EA on ST-36 on repairing the function of nitrergic nerve was fainter than that of AEA on auricular stomach point. However, there were no significant difference in the percentage of GI transit, c-kit expression and the immunohistochemical expression of ICCs in antrum after stimulations between the AEA group and ST-36 group. Several studies have found that EA on ST-36 could significantly improve the gastric emptying speed, repair the impaired gastric slow wave induced by RD and rescue ICCs networks and nNOS expression in diabetic rats [31,35,48].

It seems that AEA and EA on ST-36 could increase the GI motility through repairing the injured ICCs at different signal pathways. According to a systematic review, it has found that different acupoints have various regulation effects on GI function through different ways [49]. For example, acupuncture applied at PC-6 or ST-36 in rats significantly enhanced gastric motility, while stimulating zhongwan (CV-12) significantly suppressed gastric motility [50]. The part of mechanism is acupuncture at acupoints in limbs could promote gastric motility via a supra-spinal reflex that activated the vagal nerve fibers, while the same stimulus to the abdomen result in the reverse effect via a spinal reflex that activated sympathetic nerve fibers[51–53]. Therefore, the mechanism of AEA on regulating the GI function is needed deep researches.

## Conclusion

In conclusion, this is the first study providing the evidence for the effect of low frequency AEA on ‘stomach’ point on regulating the GI motility in STZ -induced diabetic rats. The results showed that low frequency AEA could significantly up-regulate nNOS mRNA expression and ameliorate the ICCs networks partly in gastric antrum of STZ-induced diabetic rats, which may has benefits on improving the GI motility.

## Supporting Information

S1 File Original data. (DOCX)
