# Supplementary material for: The Effects of Auricular Electro-Acupuncture on Ameliorating the Dysfunction of Interstitial Cells of Cajal Networks and nNOSmRNA Expression in Antrum of STZ-Induced Diabetic Rats
Source: PLoS One. 2016 Dec 8;11(12):e0166638. doi: 10.1371/journal.pone.0166638 (PMC5145159; doi:10.1371/journal.pone.0166638)
Supplement: S1 File — (DOCX) [file pone.0166638.s001.docx]

**Table A in S1 File GI transit rate (%)**

| Data for publish | Control Group | SEA Group | AEA Group | ST-36 Group |
| --- | --- | --- | --- | --- |
|  | 81 | 74.4 | 65.6 | 95.1 |
|  | 79.3 | 82.7 | 83.2 | 70.7 |
|  | 80.3 | 80 | 90.8 | 87.2 |
|  | 80.3 | 78.9 | 94.2 | 96.3 |
| mean | 80.2250 | 79.0000 | 83.4500 | 87.3250 |
| sd | 0.69940 | 3.45736 | 12.75761 | 11.79559 |

**Figure A in S1 File Standard curve of serum samples**

**Table B in S1 File Concentration of NO in serum samples (μM)**

| Sample* | concentration (μM) | Sample* | concentration (μM) |
| --- | --- | --- | --- |
| 1-1B | 6.09 | 5-1B | 11.28 |
| 1-2B | 4.78 | 5-3B | 8.14 |
| 1-3B | 1.38 | 5-4B | 3.12 |
| 1-4B | 9.20 | 6-1B | 1.59 |
| 2-1B | 11.57 | 6-2B | 3.82 |
| 2-2B | 17.40 | 6-3B | 3.90 |
| 2-3B | 10.81 | 6-4B | 6.80 |
| 2-4B | 7.02 | 7-1B | 3.71 |
| 2-5B | 2.54 | 7-2B | 3.17 |
| 3-1B | 6.15 | 7-3B | 4.17 |
| 3-2B | 6.22 | 7-4B | 5.06 |
| 3-3B | 4.13 | 8-1B | 3.74 |
| 3-4B | 2.67 | 8-2B | 1.36 |
| 4-1B | 3.53 | 8-3B | 2.47 |
| 4-2B | 2.63 | 8-4B | 12.62 |
| 4-3B | 16.74 |  |  |
| 4-4B | 28.92 |  |  |
| 4-5B | 2.52 |  |  |

*：1-1B、1-2B、1-3B、1-4B、8-1B、8-2B、8-3B、8-4B were from Control Group；

2-1B、2-2B、2-3B、2-4B、2-5B、3-1B、3-2B、3-3B、3-4B were from SEA Group；

4-1B、4-2B、4-3B、4-4B、4-5B、5-1B、5-3B、5-4B were from AEA Group；

6-1B、6-2B、6-3B、6-4B、7-1B、7-2B、7-3B、7-4B were from ST-Group.

**Table C in S1 File NO change in serum (μM)**

| Data for publish | Control Group | SEA Group | AEA Group | ST-36 Group |
| --- | --- | --- | --- | --- |
|  | 6.09 | 11.57 | 3.53 | 1.59 |
|  | 4.78 | 17.40 | 2.63 | 3.82 |
|  | 1.38 | 10.81 | 16.74 | 3.90 |
|  | 9.20 | 7.02 | 28.92 | 6.80 |
|  | 3.74 | 2.54 | 2.52 | 3.71 |
|  | 1.36 | 6.15 | 11.28 | 3.17 |
|  | 2.47 | 6.22 | 8.14 | 4.17 |
|  | 12.62 | 4.13 | 3.12 | 5.06 |
|  |  | 2.67 |  |  |
| mean | 5.2050 | 7.6122 | 9.6100 | 4.0275 |
| sd | 3.98116 | 4.85279 | 9.31004 | 1.49332 |

**Figure B in S1 File Standard curve of protein concentration in antrum tissue (ug/ul)**


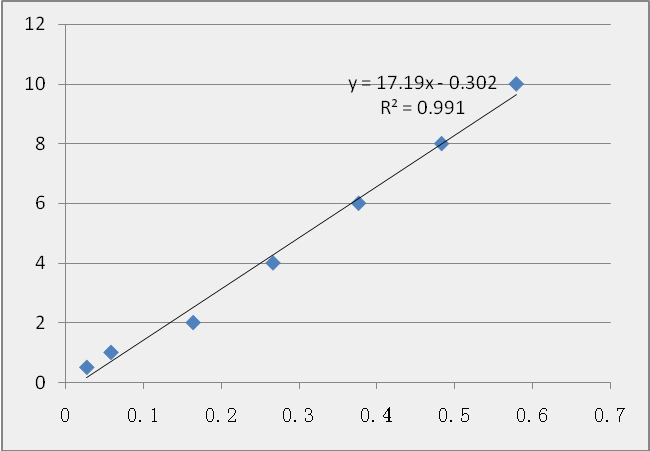


**Figure C in S1 File Band expression of GAPDH and c-Kit protein in antrum**

**
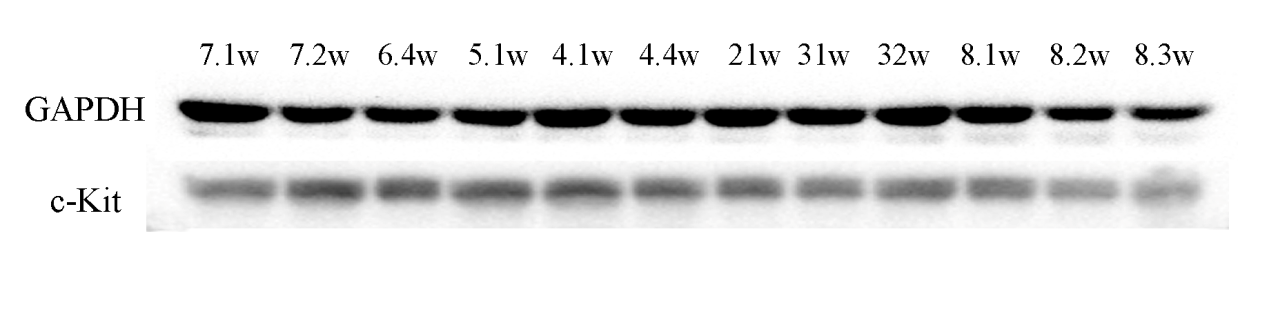
**

**Table D in S1 File The analysis of c-Kit protein expression in antrum tissue**

| Sample* | GAPDH | c-Kit | c-Kit/G |
| --- | --- | --- | --- |
| 7.1w | 6457588 | 3675563 | 0.569185 |
| 7.2w | 4982515 | 4324780 | 0.867991 |
| 6.4w | 4384379 | 3770746 | 0.860041 |
| 5.1w | 4744083 | 4265253 | 0.899068 |
| 4.1w | 5353690 | 4220571 | 0.788348 |
| 4.4w | 5332121 | 3610538 | 0.67713 |
| 21w | 5209415 | 3378954 | 0.648624 |
| 31w | 4935338 | 3010340 | 0.609956 |
| 32w | 5361362 | 3647631 | 0.680355 |
| 8.1w | 5027718 | 3229331 | 0.642306 |
| 8.2w | 3791070 | 2456481 | 0.647965 |
| 8.3w | 3618871 | 2584776 | 0.714249 |

*:7.1w、7.2w、6.4w were from ST-Group；

5.1w、4.1w、4.4w were from AEA Group；

21w、31w、32w were from SEA Group；

8.1w、8.2w、8.3w were from Control Group.

**Table E in S1 File C-Kit protein expression in antrum tissue**

| Data for publish | Control Group | SEA Group | AEA Group | ST-36 Group |
| --- | --- | --- | --- | --- |
|  | 0.642306 | 0.648624 | 0.899068 | 0.569185 |
|  | 0.647965 | 0.609956 | 0.788348 | 0.867991 |
|  | 0.714249 | 0.680355 | 0.67713 | 0.860041 |
| mean | 0.6682 | 0.6463 | 0.7882 | 0.7657 |
| sd | 0.04000 | 0.03526 | 0.11097 | 0.17027 |

**Table F in S1 File The analysis of nNOS mRNA expression in antrum tissue**

| Target | Sample* | Expression |
| --- | --- | --- |
| nNOS | 6.1w | 0.13551 |
| nNOS | 6.3w | 0.38331 |
| nNOS | 6.4w | 0.30450 |
| nNOS | 7.2w | 0.67811 |
| nNOS | 4.1w | 0.59256 |
| nNOS | 4.4w | 0.51403 |
| nNOS | 5.1w | 0.71705 |
| nNOS | 5.4w | 0.72706 |
| nNOS | 2.1w | 0.29451 |
| nNOS | 2.4w | 0.38537 |
| nNOS | 3.1w | 0.32336 |
| nNOS | 3.2w | 0.45321 |
| nNOS | 1.2w | 0.32387 |
| nNOS | 1.4w | 0.40723 |
| nNOS | 8.1w | 0.25900 |
| nNOS | 8.2w | 0.58406 |

*：6.1w、6.3w、6.4w、7.2w were from ST-Group；

4.1w、4.4w、5.1w、5.4w were from AEA Group；

2.1w、2.4w、3.1w、3.2w were from SEA Group；

1.2w、1.4w、8.1w、8.2wwere from Control Group.

**Table G in S1 File nNOS mRNA expression in antrum tissue**

| Data for publish | Control Group | SEA Group | AEA Group^＃^ | ST-36 Group |
| --- | --- | --- | --- | --- |
|  | 0.32387 | 0.29451 | 0.59256 | 0.13551 |
|  | 0.40723 | 0.38537 | 0.51403 | 0.38331 |
|  | 0.25900 | 0.32336 | 0.71705 | 0.30450 |
|  | 0.58406 | 0.45321 | 0.72706 | 0.67811 |
| mean | 0.39 | 0.36 | 0.64 | 0.38 |
| sd | 0.140759628 | 0.070465122 | 0.102654649 | 0.226766354 |
| ＃P＜0.05 VS. Control Group, SEA Group and ST-36 Group. | | | | |
